# Chloridobis(2-chloro­benz­yl)(quinolin-8-olato-κ^2^ *N*,*O*)tin(IV)

**DOI:** 10.1107/S160053681101573X

**Published:** 2011-05-07

**Authors:** Thy Chun Keng, Kong Mun Lo, Seik Weng Ng

**Affiliations:** a Department of Chemistry, University of Malaya, 50603 Kuala Lumpur, Malaysia

## Abstract

The Sn^IV^ atom in the three independent mol­ecules of the title compound, [Sn(C_7_H_6_Cl)_2_(C_9_H_6_NO)Cl], is *N*,*O*-chelated by the quinolin-8-olate anion and exists in a *cis*-C_2_SnNOCl trigonal–bipyramidal geometry; the O atom  of the anion and the two benzyl C atoms lie in the equatorial plane.

## Related literature

For the direct synthesis of the organotin chloride reactant, see: Sisido *et al.* (1961 ▶). For related structures, see: Shi & Hu (1987 ▶); Vafaee *et al.* (2010 ▶).
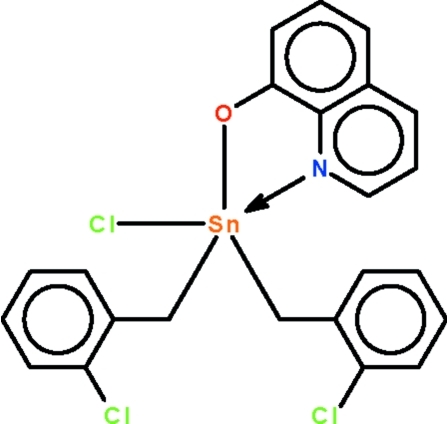


## Experimental

### 

#### Crystal data

[Sn(C_7_H_6_Cl)_2_(C_9_H_6_NO)Cl]
*M* *_r_* = 549.42
Monoclinic, 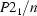

*a* = 10.2504 (1) Å
*b* = 41.2573 (6) Å
*c* = 15.3882 (2) Å
β = 100.3433 (6)°
*V* = 6401.98 (14) Å^3^
*Z* = 12
Mo *K*α radiation
μ = 1.59 mm^−1^
*T* = 100 K
0.25 × 0.20 × 0.15 mm

#### Data collection

Bruker SMART APEX diffractometer
Absorption correction: multi-scan (*SADABS*; Sheldrick, 1996 ▶) *T*_min_ = 0.692, *T*_max_ = 0.797
41105 measured reflections
14589 independent reflections
13128 reflections with *I* > 2σ(*I*)
*R*_int_ = 0.024

#### Refinement

*R*[*F* ^2^ > 2σ(*F* ^2^)] = 0.037
*wR*(*F* ^2^) = 0.086
*S* = 1.11
14589 reflections
784 parameters
H-atom parameters constrained
Δρ_max_ = 1.27 e Å^−3^
Δρ_min_ = −1.42 e Å^−3^

### 

Data collection: *APEX2* (Bruker, 2009 ▶); cell refinement: *SAINT*; data reduction: *SAINT* (Bruker, 2009 ▶); program(s) used to solve structure: *SHELXS97* (Sheldrick, 2008 ▶); program(s) used to refine structure: *SHELXL97* (Sheldrick, 2008 ▶); molecular graphics: *X-SEED* (Barbour, 2001 ▶); software used to prepare material for publication: *publCIF* (Westrip, 2010 ▶).

## Supplementary Material

Crystal structure: contains datablocks global, I. DOI: 10.1107/S160053681101573X/bt5528sup1.cif

Structure factors: contains datablocks I. DOI: 10.1107/S160053681101573X/bt5528Isup2.hkl

Additional supplementary materials:  crystallographic information; 3D view; checkCIF report

## Figures and Tables

**Fig. 1.**
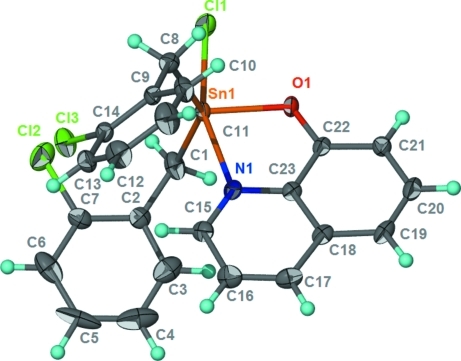
Anisotropic displacement ellipsoid plot (Barbour, 2001) of molecule 1 of SnCl(C7H6Cl)2(C9H6NO) at the 70% probability level; hydrogen atoms are drawn as spheres of arbitrary radius.

**Fig. 2.**
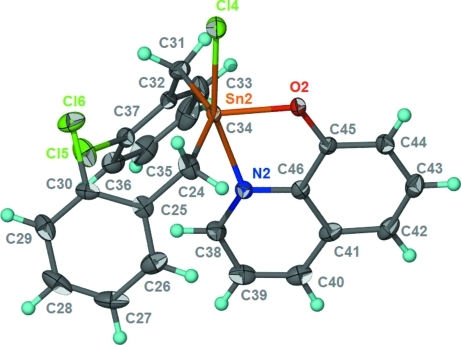
Anisotropic displacement ellipsoid plot (Barbour, 2001) of molecule 2 of SnCl(C7H6Cl)2(C9H6NO) at the 70% probability level; hydrogen atoms are drawn as spheres of arbitrary radius.

**Fig. 3.**
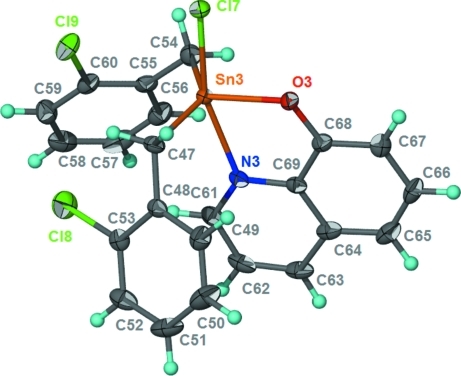
Anisotropic displacement ellipsoid plot (Barbour, 2001) of molecule 3 of SnCl(C7H6Cl)2(C9H6NO) at the 70% probability level; hydrogen atoms are drawn as spheres of arbitrary radius.

**Fig. 4.**
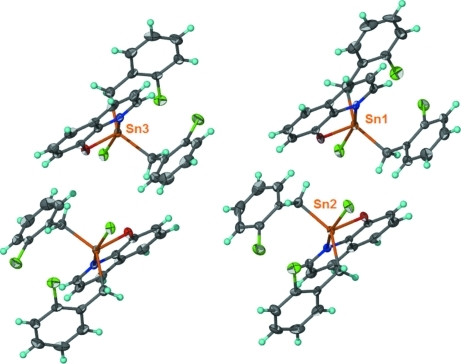
Positions of molecules 1 and 2, and the inversion-related pair of molecule 3.

## supplementary crystallographic information

### Comment

8-Hydroxyquinoline forms a large number of derivatives with organotin systems; when the reaction is carried out with diorganotin dichlorides, in some cases only one chloride is displaced whereas in other cases both are displaced. No base is added to abstract the acid hydroxy proton. In the present study, the reaction of 8-hydroxyquinoline with bis(2-chlorobenzyl)tin dichloride affords SnCl(C_7_H_6_Cl)_2_(C_9_H_6_NO) (Scheme I). The Sn^IV^ atom in the three independent molecules *N*,*O*-chelated by the quinolinato group and it exists in a *cis*-C_2_SnNOCl trigonal bipyramidal geometry; the O atom lies in the equatorial plane and the Cl and N atoms comprise the axial sites (Figs. 1, 2, 3). The molecules are positioned such that two are disposed about a false-center of inversion (Fig. 2). There are no Sn···Cl contacts and the distortion of geometry from the idealized trigonal bipyramid is minimal (14.7% in the first molecule, 13.7% in the second and only 9.9% in the third).

In the diethytin derivative, an intermolecule Sn···Cl interaction raises the coordination number of Sn to six in the chain structure (Shi & Hu, 1987). On the other hand, the methylphenyltin derivative also has Sn in a six-coordinate environment, but the higher coordination number arises from bridging by the quinolinate ion that results in a dinuclear molecule (Vafaee *et al.*, 2010).

### Experimental

The diorganotin dichloride was synthesized by the direct reaction of 2-chlorobenzyl chloride and metallic tin according to a literature procedure (Sisido *et al.*, 1961). The diorganotin dichloride (0.44 g, 1 mmol) and 8-hydroxyquinoline (0.15 g, 1 mmol) were dissolved in chloroform (50 ml) to give a yellow solution. The solution was filtered and the solvent allowed to evaporate to yield yellow crystals.

### Refinement

H-atoms were placed in calculated positions (C—H 0.95 to 0.99 Å) and were included in the refinement in the riding model approximation, with *U*(H) set to 1.2 times *U*_eq_(C).

The final difference Fourier map had a peak in the vicinity of Sn1 as well as a hole in the vicinity of the same atom.

The second parameter of the weighting scheme is rather large; however, as lowering this number to less than 10 had only a minor effect on the refinement with regard to the peak/hole. This high value was retained.

### Crystal data

**Table:**

[Sn(C_7_H_6_Cl)_2_(C_9_H_6_NO)Cl]	*F*(000) = 3264
*M**_r_* = 549.42	*D*_x_ = 1.710 Mg m^−^^3^
Monoclinic, *P*2_1_/*n*	Mo *K*α radiation, λ = 0.71073 Å
Hall symbol: -P 2yn	Cell parameters from 9252 reflections
*a* = 10.2504 (1) Å	θ = 2.4–28.3°
*b* = 41.2573 (6) Å	µ = 1.59 mm^−^^1^
*c* = 15.3882 (2) Å	*T* = 100 K
β = 100.3433 (6)°	Block, colorless
*V* = 6401.98 (14) Å^3^	0.25 × 0.20 × 0.15 mm
*Z* = 12	

### Data collection

**Table:**

Bruker SMART APEX diffractometer	14589 independent reflections
Radiation source: fine-focus sealed tube	13128 reflections with *I* > 2σ(*I*)
graphite	*R*_int_ = 0.024
ω scans	θ_max_ = 27.5°, θ_min_ = 1.0°
Absorption correction: multi-scan (*SADABS*; Sheldrick, 1996)	*h* = −13→13
*T*_min_ = 0.692, *T*_max_ = 0.797	*k* = −53→40
41105 measured reflections	*l* = −19→19

### Refinement

**Table:**

Refinement on *F*^2^	Primary atom site location: structure-invariant direct methods
Least-squares matrix: full	Secondary atom site location: difference Fourier map
*R*[*F*^2^ > 2σ(*F*^2^)] = 0.037	Hydrogen site location: inferred from neighbouring sites
*wR*(*F*^2^) = 0.086	H-atom parameters constrained
*S* = 1.11	*w* = 1/[σ^2^(*F*_o_^2^) + (0.0318*P*)^2^ + 14.3485*P*] where *P* = (*F*_o_^2^ + 2*F*_c_^2^)/3
14589 reflections	(Δ/σ)_max_ = 0.001
784 parameters	Δρ_max_ = 1.27 e Å^−^^3^
0 restraints	Δρ_min_ = −1.42 e Å^−^^3^

### Special details

**Table:**

Geometry. All e.s.d.'s (except the e.s.d. in the dihedral angle between two l.s. planes) are estimated using the full covariance matrix. The cell e.s.d.'s are taken into account individually in the estimation of e.s.d.'s in distances, angles and torsion angles; correlations between e.s.d.'s in cell parameters are only used when they are defined by crystal symmetry. An approximate (isotropic) treatment of cell e.s.d.'s is used for estimating e.s.d.'s involving l.s. planes.

### Fractional atomic coordinates and isotropic or equivalent isotropic displacement parameters (Å^2^)

**Table:**

	*x*	*y*	*z*	*U*_iso_*/*U*_eq_	
Sn1	0.51891 (2)	0.342454 (5)	0.664659 (14)	0.01383 (5)	
Sn2	0.51124 (2)	0.325014 (5)	0.306586 (14)	0.01375 (5)	
Sn3	0.49861 (2)	0.005273 (5)	0.701013 (14)	0.01335 (5)	
Cl1	0.35889 (8)	0.30078 (2)	0.60004 (6)	0.02297 (17)	
Cl2	0.28721 (9)	0.35808 (3)	0.86345 (6)	0.0307 (2)	
Cl3	0.37714 (9)	0.41474 (2)	0.73481 (6)	0.02583 (18)	
Cl4	0.66348 (8)	0.36906 (2)	0.36650 (6)	0.02405 (18)	
Cl5	0.65041 (10)	0.24788 (2)	0.24180 (6)	0.0301 (2)	
Cl6	0.76770 (9)	0.31831 (2)	0.15379 (6)	0.02775 (19)	
Cl7	0.33400 (8)	−0.036560 (19)	0.64966 (6)	0.02048 (16)	
Cl8	0.38458 (10)	0.09956 (2)	0.74145 (6)	0.0296 (2)	
Cl9	0.26222 (8)	0.02390 (2)	0.83358 (5)	0.02545 (18)	
O1	0.6547 (2)	0.31709 (5)	0.60960 (14)	0.0158 (4)	
O2	0.3754 (2)	0.34906 (5)	0.36442 (14)	0.0160 (4)	
O3	0.6230 (2)	−0.01859 (5)	0.63342 (15)	0.0172 (5)	
N1	0.7171 (3)	0.37103 (6)	0.70561 (17)	0.0152 (5)	
N2	0.3191 (3)	0.29384 (6)	0.27310 (17)	0.0149 (5)	
N3	0.6961 (3)	0.03576 (6)	0.72578 (17)	0.0155 (5)	
C1	0.5196 (4)	0.32487 (8)	0.7971 (2)	0.0228 (7)	
H1A	0.4312	0.3154	0.7984	0.027*	
H1B	0.5847	0.3070	0.8083	0.027*	
C2	0.5502 (3)	0.34804 (8)	0.8716 (2)	0.0181 (7)	
C3	0.6818 (4)	0.35385 (10)	0.9111 (3)	0.0296 (8)	
H3	0.7503	0.3422	0.8907	0.036*	
C4	0.7155 (4)	0.37575 (11)	0.9782 (3)	0.0390 (11)	
H4	0.8062	0.3792	1.0030	0.047*	
C5	0.6192 (5)	0.39269 (10)	1.0095 (2)	0.0403 (12)	
H5	0.6430	0.4080	1.0557	0.048*	
C6	0.4858 (5)	0.38745 (9)	0.9736 (2)	0.0321 (9)	
H6	0.4180	0.3989	0.9955	0.039*	
C7	0.4539 (3)	0.36523 (8)	0.9056 (2)	0.0197 (7)	
C8	0.4283 (3)	0.37879 (8)	0.5727 (2)	0.0195 (7)	
H8A	0.4374	0.3717	0.5126	0.023*	
H8B	0.3323	0.3792	0.5747	0.023*	
C9	0.4790 (3)	0.41255 (8)	0.5851 (2)	0.0165 (6)	
C10	0.5479 (4)	0.42694 (9)	0.5246 (2)	0.0227 (7)	
H10	0.5657	0.4146	0.4759	0.027*	
C11	0.5910 (4)	0.45888 (10)	0.5344 (3)	0.0302 (8)	
H11	0.6367	0.4682	0.4921	0.036*	
C12	0.5676 (4)	0.47723 (9)	0.6057 (2)	0.0265 (8)	
H12	0.5977	0.4990	0.6123	0.032*	
C13	0.5003 (3)	0.46368 (8)	0.6667 (2)	0.0210 (7)	
H13	0.4830	0.4760	0.7155	0.025*	
C14	0.4584 (3)	0.43187 (8)	0.6559 (2)	0.0161 (6)	
C15	0.7455 (3)	0.39726 (8)	0.7550 (2)	0.0178 (6)	
H15	0.6763	0.4076	0.7781	0.021*	
C16	0.8738 (3)	0.41027 (8)	0.7745 (2)	0.0221 (7)	
H16	0.8910	0.4289	0.8109	0.027*	
C17	0.9739 (3)	0.39583 (8)	0.7406 (2)	0.0214 (7)	
H17	1.0609	0.4046	0.7530	0.026*	
C18	0.9483 (3)	0.36779 (8)	0.6871 (2)	0.0162 (6)	
C19	1.0442 (3)	0.35066 (8)	0.6496 (2)	0.0193 (7)	
H19	1.1330	0.3583	0.6577	0.023*	
C20	1.0088 (3)	0.32306 (9)	0.6017 (2)	0.0211 (7)	
H20	1.0742	0.3117	0.5772	0.025*	
C21	0.8782 (3)	0.31108 (8)	0.5878 (2)	0.0171 (6)	
H21	0.8569	0.2917	0.5549	0.020*	
C22	0.7806 (3)	0.32750 (8)	0.62184 (19)	0.0144 (6)	
C23	0.8163 (3)	0.35615 (8)	0.67214 (19)	0.0144 (6)	
C24	0.4908 (4)	0.34026 (8)	0.1706 (2)	0.0223 (7)	
H24A	0.4032	0.3508	0.1536	0.027*	
H24B	0.5592	0.3569	0.1670	0.027*	
C25	0.5024 (3)	0.31453 (8)	0.1037 (2)	0.0165 (6)	
C26	0.3891 (4)	0.30107 (9)	0.0511 (2)	0.0248 (8)	
H26	0.3037	0.3085	0.0575	0.030*	
C27	0.4002 (4)	0.27714 (10)	−0.0099 (2)	0.0302 (9)	
H27	0.3223	0.2685	−0.0451	0.036*	
C28	0.5223 (4)	0.26566 (9)	−0.0205 (2)	0.0300 (9)	
H28	0.5287	0.2490	−0.0621	0.036*	
C29	0.6358 (4)	0.27858 (9)	0.0299 (2)	0.0250 (8)	
H29	0.7207	0.2709	0.0232	0.030*	
C30	0.6244 (3)	0.30281 (8)	0.0901 (2)	0.0179 (6)	
C31	0.6184 (3)	0.29074 (8)	0.3978 (2)	0.0211 (7)	
H31A	0.7108	0.2902	0.3871	0.025*	
H31B	0.6217	0.2995	0.4580	0.025*	
C32	0.5725 (3)	0.25673 (8)	0.3986 (2)	0.0165 (6)	
C33	0.5197 (4)	0.24448 (10)	0.4697 (2)	0.0270 (8)	
H33	0.5067	0.2588	0.5158	0.032*	
C34	0.4858 (4)	0.21223 (11)	0.4750 (3)	0.0345 (9)	
H34	0.4518	0.2046	0.5247	0.041*	
C35	0.5013 (4)	0.19110 (10)	0.4077 (3)	0.0313 (9)	
H35	0.4785	0.1689	0.4115	0.038*	
C36	0.5498 (4)	0.20224 (9)	0.3353 (2)	0.0244 (7)	
H36	0.5591	0.1880	0.2883	0.029*	
C37	0.5848 (3)	0.23456 (8)	0.3323 (2)	0.0174 (6)	
C38	0.2936 (3)	0.26708 (8)	0.2257 (2)	0.0183 (6)	
H38	0.3630	0.2573	0.2014	0.022*	
C39	0.1678 (4)	0.25257 (8)	0.2101 (2)	0.0227 (7)	
H39	0.1524	0.2334	0.1754	0.027*	
C40	0.0673 (3)	0.26633 (8)	0.2456 (2)	0.0217 (7)	
H40	−0.0182	0.2566	0.2355	0.026*	
C41	0.0902 (3)	0.29479 (8)	0.2971 (2)	0.0185 (7)	
C42	−0.0053 (3)	0.31081 (9)	0.3369 (2)	0.0218 (7)	
H42	−0.0920	0.3020	0.3322	0.026*	
C43	0.0271 (3)	0.33903 (9)	0.3824 (2)	0.0218 (7)	
H43	−0.0385	0.3498	0.4081	0.026*	
C44	0.1552 (3)	0.35252 (9)	0.3923 (2)	0.0193 (7)	
H44	0.1746	0.3722	0.4240	0.023*	
C45	0.2524 (3)	0.33724 (8)	0.35614 (19)	0.0144 (6)	
C46	0.2201 (3)	0.30805 (8)	0.3085 (2)	0.0151 (6)	
C47	0.3890 (3)	0.04155 (8)	0.6179 (2)	0.0177 (6)	
H47A	0.3528	0.0315	0.5602	0.021*	
H47B	0.3127	0.0483	0.6450	0.021*	
C48	0.4642 (3)	0.07106 (8)	0.6015 (2)	0.0158 (6)	
C49	0.5337 (3)	0.07149 (9)	0.5316 (2)	0.0232 (7)	
H49	0.5317	0.0530	0.4947	0.028*	
C50	0.6058 (4)	0.09847 (11)	0.5153 (3)	0.0334 (9)	
H50	0.6516	0.0985	0.4668	0.040*	
C51	0.6113 (4)	0.12534 (10)	0.5691 (3)	0.0311 (9)	
H51	0.6623	0.1436	0.5582	0.037*	
C52	0.5433 (4)	0.12588 (9)	0.6389 (2)	0.0244 (7)	
H52	0.5463	0.1444	0.6757	0.029*	
C53	0.4706 (3)	0.09875 (8)	0.6540 (2)	0.0175 (6)	
C54	0.5296 (4)	−0.00778 (8)	0.8390 (2)	0.0211 (7)	
H54A	0.4609	−0.0237	0.8482	0.025*	
H54B	0.6170	−0.0185	0.8551	0.025*	
C55	0.5246 (3)	0.02044 (8)	0.8991 (2)	0.0182 (7)	
C56	0.6380 (4)	0.03202 (9)	0.9556 (2)	0.0238 (7)	
H56	0.7202	0.0212	0.9572	0.029*	
C57	0.6332 (4)	0.05867 (10)	1.0089 (2)	0.0292 (9)	
H57	0.7115	0.0658	1.0467	0.035*	
C58	0.5157 (4)	0.07504 (9)	1.0076 (2)	0.0280 (8)	
H58	0.5136	0.0936	1.0437	0.034*	
C59	0.3999 (4)	0.06433 (9)	0.9535 (2)	0.0234 (7)	
H59	0.3181	0.0753	0.9524	0.028*	
C60	0.4070 (3)	0.03732 (8)	0.9013 (2)	0.0178 (7)	
C61	0.7283 (3)	0.06276 (8)	0.7710 (2)	0.0194 (7)	
H61	0.6642	0.0728	0.7996	0.023*	
C62	0.8548 (4)	0.07705 (8)	0.7782 (2)	0.0243 (7)	
H62	0.8755	0.0964	0.8115	0.029*	
C63	0.9477 (3)	0.06275 (9)	0.7366 (2)	0.0236 (7)	
H63	1.0329	0.0723	0.7408	0.028*	
C64	0.9170 (3)	0.03384 (8)	0.6874 (2)	0.0193 (7)	
C65	1.0060 (3)	0.01673 (9)	0.6442 (2)	0.0234 (7)	
H65	1.0927	0.0250	0.6447	0.028*	
C66	0.9673 (3)	−0.01177 (9)	0.6017 (2)	0.0217 (7)	
H66	1.0287	−0.0234	0.5741	0.026*	
C67	0.8383 (3)	−0.02427 (9)	0.5979 (2)	0.0191 (7)	
H67	0.8140	−0.0441	0.5680	0.023*	
C68	0.7474 (3)	−0.00779 (8)	0.6374 (2)	0.0161 (6)	
C69	0.7877 (3)	0.02121 (8)	0.6840 (2)	0.0153 (6)	

### Atomic displacement parameters (Å^2^)

**Table:**

	*U*^11^	*U*^22^	*U*^33^	*U*^12^	*U*^13^	*U*^23^
Sn1	0.01268 (10)	0.01196 (11)	0.01760 (10)	0.00043 (8)	0.00474 (8)	−0.00269 (8)
Sn2	0.01284 (10)	0.01225 (11)	0.01672 (10)	−0.00010 (8)	0.00417 (8)	−0.00330 (8)
Sn3	0.01349 (10)	0.01096 (11)	0.01619 (10)	−0.00032 (8)	0.00426 (8)	0.00004 (8)
Cl1	0.0155 (4)	0.0190 (4)	0.0347 (4)	−0.0035 (3)	0.0052 (3)	−0.0078 (3)
Cl2	0.0187 (4)	0.0405 (6)	0.0346 (5)	−0.0005 (4)	0.0093 (4)	0.0044 (4)
Cl3	0.0314 (5)	0.0270 (5)	0.0231 (4)	0.0010 (4)	0.0154 (3)	0.0016 (3)
Cl4	0.0185 (4)	0.0203 (4)	0.0347 (4)	−0.0064 (3)	0.0084 (3)	−0.0108 (3)
Cl5	0.0408 (5)	0.0321 (5)	0.0213 (4)	0.0002 (4)	0.0160 (4)	0.0001 (3)
Cl6	0.0199 (4)	0.0336 (5)	0.0279 (4)	−0.0079 (4)	−0.0007 (3)	0.0006 (4)
Cl7	0.0163 (4)	0.0156 (4)	0.0305 (4)	−0.0040 (3)	0.0067 (3)	−0.0021 (3)
Cl8	0.0376 (5)	0.0311 (5)	0.0222 (4)	0.0009 (4)	0.0114 (4)	−0.0044 (3)
Cl9	0.0158 (4)	0.0385 (5)	0.0219 (4)	−0.0020 (3)	0.0028 (3)	−0.0009 (3)
O1	0.0125 (11)	0.0147 (11)	0.0211 (11)	0.0009 (8)	0.0054 (9)	−0.0039 (9)
O2	0.0141 (11)	0.0158 (12)	0.0186 (11)	0.0012 (9)	0.0039 (9)	−0.0045 (9)
O3	0.0140 (11)	0.0151 (12)	0.0232 (11)	−0.0024 (9)	0.0057 (9)	−0.0038 (9)
N1	0.0151 (13)	0.0127 (13)	0.0172 (12)	0.0038 (10)	0.0013 (10)	0.0017 (10)
N2	0.0160 (13)	0.0134 (13)	0.0150 (12)	0.0012 (10)	0.0021 (10)	−0.0002 (10)
N3	0.0173 (13)	0.0112 (13)	0.0174 (12)	0.0001 (10)	0.0014 (10)	0.0016 (10)
C1	0.0310 (19)	0.0171 (17)	0.0235 (17)	0.0012 (14)	0.0134 (15)	0.0009 (13)
C2	0.0188 (16)	0.0215 (17)	0.0148 (14)	−0.0005 (13)	0.0052 (12)	0.0041 (12)
C3	0.0224 (18)	0.036 (2)	0.0301 (19)	−0.0018 (16)	0.0036 (15)	0.0105 (16)
C4	0.037 (2)	0.045 (3)	0.029 (2)	−0.021 (2)	−0.0089 (18)	0.0123 (18)
C5	0.075 (3)	0.030 (2)	0.0127 (16)	−0.031 (2)	−0.0031 (19)	−0.0002 (15)
C6	0.055 (3)	0.023 (2)	0.0227 (17)	−0.0014 (18)	0.0174 (18)	−0.0007 (15)
C7	0.0234 (17)	0.0186 (17)	0.0173 (15)	−0.0042 (13)	0.0039 (13)	−0.0003 (12)
C8	0.0173 (16)	0.0193 (17)	0.0212 (16)	0.0018 (13)	0.0015 (13)	−0.0023 (13)
C9	0.0167 (15)	0.0180 (17)	0.0141 (14)	0.0066 (13)	0.0009 (12)	0.0005 (12)
C10	0.0277 (19)	0.0236 (19)	0.0184 (16)	0.0060 (14)	0.0089 (14)	0.0005 (13)
C11	0.038 (2)	0.028 (2)	0.0294 (19)	0.0008 (17)	0.0174 (17)	0.0054 (15)
C12	0.034 (2)	0.0150 (17)	0.0311 (19)	−0.0002 (15)	0.0071 (16)	0.0019 (14)
C13	0.0235 (17)	0.0202 (17)	0.0191 (15)	0.0059 (14)	0.0035 (13)	−0.0035 (13)
C14	0.0160 (15)	0.0187 (17)	0.0140 (14)	0.0032 (12)	0.0039 (12)	0.0017 (12)
C15	0.0205 (16)	0.0126 (15)	0.0192 (15)	0.0025 (12)	0.0003 (13)	0.0010 (12)
C16	0.0259 (18)	0.0138 (16)	0.0255 (17)	−0.0007 (14)	0.0013 (14)	−0.0003 (13)
C17	0.0204 (17)	0.0194 (17)	0.0228 (16)	−0.0057 (13)	−0.0005 (13)	0.0039 (13)
C18	0.0151 (15)	0.0166 (16)	0.0165 (14)	0.0001 (12)	0.0016 (12)	0.0059 (12)
C19	0.0124 (15)	0.0230 (18)	0.0227 (16)	0.0018 (13)	0.0039 (13)	0.0078 (13)
C20	0.0161 (16)	0.031 (2)	0.0170 (15)	0.0067 (14)	0.0052 (13)	0.0051 (13)
C21	0.0169 (15)	0.0199 (17)	0.0146 (14)	0.0022 (13)	0.0034 (12)	0.0002 (12)
C22	0.0129 (14)	0.0176 (16)	0.0127 (13)	0.0021 (12)	0.0027 (11)	0.0040 (11)
C23	0.0153 (15)	0.0148 (16)	0.0134 (14)	−0.0008 (12)	0.0034 (12)	0.0029 (11)
C24	0.0309 (19)	0.0156 (17)	0.0223 (16)	0.0018 (14)	0.0100 (14)	0.0020 (13)
C25	0.0247 (17)	0.0093 (15)	0.0159 (14)	0.0019 (12)	0.0048 (13)	0.0068 (11)
C26	0.0214 (17)	0.026 (2)	0.0249 (17)	−0.0017 (14)	−0.0011 (14)	0.0080 (14)
C27	0.038 (2)	0.028 (2)	0.0204 (17)	−0.0175 (17)	−0.0062 (15)	0.0017 (15)
C28	0.053 (3)	0.0219 (19)	0.0163 (16)	−0.0087 (17)	0.0086 (16)	−0.0043 (14)
C29	0.033 (2)	0.0232 (19)	0.0227 (17)	−0.0016 (15)	0.0143 (15)	−0.0028 (14)
C30	0.0211 (16)	0.0187 (17)	0.0133 (14)	−0.0068 (13)	0.0014 (12)	0.0012 (12)
C31	0.0188 (16)	0.0184 (17)	0.0235 (17)	0.0036 (13)	−0.0028 (13)	−0.0024 (13)
C32	0.0153 (15)	0.0184 (17)	0.0149 (14)	0.0057 (12)	0.0000 (12)	−0.0010 (12)
C33	0.030 (2)	0.035 (2)	0.0174 (16)	0.0131 (16)	0.0094 (14)	0.0024 (14)
C34	0.028 (2)	0.042 (3)	0.038 (2)	0.0057 (17)	0.0169 (17)	0.0187 (18)
C35	0.027 (2)	0.021 (2)	0.046 (2)	0.0004 (15)	0.0058 (17)	0.0113 (17)
C36	0.0228 (18)	0.0191 (18)	0.0293 (18)	0.0019 (14)	−0.0004 (15)	−0.0026 (14)
C37	0.0175 (16)	0.0223 (17)	0.0130 (14)	0.0018 (13)	0.0046 (12)	0.0014 (12)
C38	0.0210 (16)	0.0137 (16)	0.0196 (15)	0.0014 (13)	0.0019 (13)	−0.0010 (12)
C39	0.0283 (19)	0.0137 (17)	0.0240 (17)	−0.0061 (14)	−0.0007 (14)	0.0015 (13)
C40	0.0217 (17)	0.0200 (18)	0.0219 (16)	−0.0050 (14)	−0.0003 (13)	0.0060 (13)
C41	0.0168 (16)	0.0214 (17)	0.0163 (15)	−0.0025 (13)	0.0004 (12)	0.0054 (12)
C42	0.0135 (16)	0.030 (2)	0.0224 (16)	−0.0001 (14)	0.0041 (13)	0.0094 (14)
C43	0.0182 (16)	0.031 (2)	0.0169 (15)	0.0054 (14)	0.0066 (13)	0.0055 (13)
C44	0.0198 (16)	0.0226 (18)	0.0152 (15)	0.0046 (13)	0.0024 (12)	−0.0004 (12)
C45	0.0142 (15)	0.0171 (16)	0.0116 (13)	0.0017 (12)	0.0016 (11)	0.0027 (11)
C46	0.0168 (15)	0.0141 (16)	0.0141 (14)	0.0022 (12)	0.0015 (12)	0.0026 (11)
C47	0.0178 (16)	0.0124 (16)	0.0221 (16)	−0.0021 (12)	0.0011 (13)	0.0005 (12)
C48	0.0144 (15)	0.0137 (16)	0.0178 (15)	0.0013 (12)	−0.0009 (12)	0.0026 (12)
C49	0.0221 (17)	0.0271 (19)	0.0210 (16)	0.0019 (14)	0.0061 (14)	0.0008 (14)
C50	0.031 (2)	0.043 (3)	0.0288 (19)	−0.0078 (18)	0.0110 (16)	0.0082 (17)
C51	0.027 (2)	0.029 (2)	0.035 (2)	−0.0109 (16)	0.0002 (16)	0.0112 (16)
C52	0.0253 (18)	0.0165 (17)	0.0271 (17)	−0.0022 (14)	−0.0066 (14)	0.0016 (14)
C53	0.0169 (16)	0.0192 (17)	0.0150 (14)	0.0027 (12)	−0.0006 (12)	0.0018 (12)
C54	0.0230 (17)	0.0200 (18)	0.0213 (16)	0.0026 (13)	0.0071 (14)	0.0026 (13)
C55	0.0197 (16)	0.0187 (17)	0.0165 (15)	−0.0010 (13)	0.0040 (12)	0.0092 (12)
C56	0.0206 (17)	0.031 (2)	0.0202 (16)	0.0000 (14)	0.0048 (13)	0.0065 (14)
C57	0.029 (2)	0.041 (2)	0.0172 (16)	−0.0162 (17)	0.0018 (14)	0.0013 (15)
C58	0.044 (2)	0.0230 (19)	0.0194 (16)	−0.0084 (16)	0.0115 (16)	−0.0030 (14)
C59	0.0303 (19)	0.0229 (19)	0.0186 (16)	0.0043 (15)	0.0089 (14)	0.0000 (13)
C60	0.0174 (16)	0.0238 (18)	0.0125 (14)	−0.0018 (13)	0.0036 (12)	0.0027 (12)
C61	0.0209 (17)	0.0146 (16)	0.0202 (15)	0.0007 (13)	−0.0033 (13)	0.0010 (12)
C62	0.0263 (18)	0.0126 (17)	0.0299 (18)	−0.0026 (14)	−0.0062 (15)	0.0027 (13)
C63	0.0194 (17)	0.0198 (18)	0.0283 (18)	−0.0068 (14)	−0.0042 (14)	0.0072 (14)
C64	0.0165 (16)	0.0204 (17)	0.0191 (15)	−0.0039 (13)	−0.0022 (12)	0.0066 (13)
C65	0.0160 (16)	0.034 (2)	0.0205 (16)	−0.0020 (14)	0.0039 (13)	0.0086 (14)
C66	0.0166 (16)	0.034 (2)	0.0164 (15)	0.0036 (14)	0.0069 (13)	0.0043 (13)
C67	0.0197 (16)	0.0229 (18)	0.0143 (14)	0.0017 (13)	0.0020 (12)	−0.0001 (12)
C68	0.0146 (15)	0.0171 (16)	0.0159 (14)	−0.0008 (12)	0.0014 (12)	0.0032 (12)
C69	0.0173 (15)	0.0127 (15)	0.0154 (14)	−0.0005 (12)	0.0014 (12)	0.0035 (11)

### Geometric parameters (Å, °)

**Table:**

Sn1—O1	2.043 (2)	C26—H26	0.9500
Sn1—C8	2.154 (3)	C27—C28	1.375 (6)
Sn1—C1	2.162 (3)	C27—H27	0.9500
Sn1—N1	2.335 (3)	C28—C29	1.384 (5)
Sn1—Cl1	2.4596 (8)	C28—H28	0.9500
Sn2—O2	2.039 (2)	C29—C30	1.382 (5)
Sn2—C31	2.150 (3)	C29—H29	0.9500
Sn2—C24	2.159 (3)	C31—C32	1.481 (5)
Sn2—N2	2.331 (3)	C31—H31A	0.9900
Sn2—Cl4	2.4638 (8)	C31—H31B	0.9900
Sn3—O3	2.038 (2)	C32—C37	1.393 (4)
Sn3—C47	2.150 (3)	C32—C33	1.399 (5)
Sn3—C54	2.159 (3)	C33—C34	1.381 (6)
Sn3—N3	2.355 (3)	C33—H33	0.9500
Sn3—Cl7	2.4437 (8)	C34—C35	1.384 (6)
Cl2—C7	1.740 (4)	C34—H34	0.9500
Cl3—C14	1.741 (3)	C35—C36	1.378 (5)
Cl5—C37	1.741 (3)	C35—H35	0.9500
Cl6—C30	1.735 (3)	C36—C37	1.384 (5)
Cl8—C53	1.736 (3)	C36—H36	0.9500
Cl9—C60	1.743 (3)	C38—C39	1.403 (5)
O1—C22	1.341 (4)	C38—H38	0.9500
O2—C45	1.337 (4)	C39—C40	1.373 (5)
O3—C68	1.342 (4)	C39—H39	0.9500
N1—C15	1.325 (4)	C40—C41	1.412 (5)
N1—C23	1.365 (4)	C40—H40	0.9500
N2—C38	1.323 (4)	C41—C42	1.410 (5)
N2—C46	1.367 (4)	C41—C46	1.421 (4)
N3—C61	1.324 (4)	C42—C43	1.368 (5)
N3—C69	1.368 (4)	C42—H42	0.9500
C1—C2	1.483 (5)	C43—C44	1.408 (5)
C1—H1A	0.9900	C43—H43	0.9500
C1—H1B	0.9900	C44—C45	1.378 (4)
C2—C7	1.391 (5)	C44—H44	0.9500
C2—C3	1.397 (5)	C45—C46	1.418 (4)
C3—C4	1.368 (6)	C47—C48	1.486 (4)
C3—H3	0.9500	C47—H47A	0.9900
C4—C5	1.366 (7)	C47—H47B	0.9900
C4—H4	0.9500	C48—C49	1.393 (5)
C5—C6	1.397 (6)	C48—C53	1.394 (5)
C5—H5	0.9500	C49—C50	1.384 (5)
C6—C7	1.386 (5)	C49—H49	0.9500
C6—H6	0.9500	C50—C51	1.379 (6)
C8—C9	1.487 (5)	C50—H50	0.9500
C8—H8A	0.9900	C51—C52	1.381 (5)
C8—H8B	0.9900	C51—H51	0.9500
C9—C14	1.396 (4)	C52—C53	1.388 (5)
C9—C10	1.397 (5)	C52—H52	0.9500
C10—C11	1.389 (5)	C54—C55	1.493 (5)
C10—H10	0.9500	C54—H54A	0.9900
C11—C12	1.388 (5)	C54—H54B	0.9900
C11—H11	0.9500	C55—C60	1.398 (5)
C12—C13	1.379 (5)	C55—C56	1.405 (5)
C12—H12	0.9500	C56—C57	1.377 (5)
C13—C14	1.382 (5)	C56—H56	0.9500
C13—H13	0.9500	C57—C58	1.379 (6)
C15—C16	1.402 (5)	C57—H57	0.9500
C15—H15	0.9500	C58—C59	1.394 (5)
C16—C17	1.369 (5)	C58—H58	0.9500
C16—H16	0.9500	C59—C60	1.383 (5)
C17—C18	1.416 (5)	C59—H59	0.9500
C17—H17	0.9500	C61—C62	1.411 (5)
C18—C19	1.415 (5)	C61—H61	0.9500
C18—C23	1.415 (4)	C62—C63	1.372 (5)
C19—C20	1.370 (5)	C62—H62	0.9500
C19—H19	0.9500	C63—C64	1.417 (5)
C20—C21	1.407 (5)	C63—H63	0.9500
C20—H20	0.9500	C64—C65	1.411 (5)
C21—C22	1.387 (4)	C64—C69	1.417 (4)
C21—H21	0.9500	C65—C66	1.369 (5)
C22—C23	1.424 (4)	C65—H65	0.9500
C24—C25	1.498 (5)	C66—C67	1.410 (5)
C24—H24A	0.9900	C66—H66	0.9500
C24—H24B	0.9900	C67—C68	1.380 (4)
C25—C30	1.392 (5)	C67—H67	0.9500
C25—C26	1.405 (5)	C68—C69	1.418 (4)
C26—C27	1.381 (5)		
			
O1—Sn1—C8	109.26 (11)	C29—C28—H28	120.2
O1—Sn1—C1	109.37 (11)	C30—C29—C28	119.4 (3)
C8—Sn1—C1	141.32 (13)	C30—C29—H29	120.3
O1—Sn1—N1	75.30 (9)	C28—C29—H29	120.3
C8—Sn1—N1	94.45 (11)	C29—C30—C25	122.6 (3)
C1—Sn1—N1	93.41 (12)	C29—C30—Cl6	118.7 (3)
O1—Sn1—Cl1	85.92 (7)	C25—C30—Cl6	118.7 (3)
C8—Sn1—Cl1	92.48 (9)	C32—C31—Sn2	120.2 (2)
C1—Sn1—Cl1	92.04 (10)	C32—C31—H31A	107.3
N1—Sn1—Cl1	161.20 (7)	Sn2—C31—H31A	107.3
O2—Sn2—C31	110.12 (11)	C32—C31—H31B	107.3
O2—Sn2—C24	109.04 (11)	Sn2—C31—H31B	107.3
C31—Sn2—C24	140.67 (13)	H31A—C31—H31B	106.9
O2—Sn2—N2	75.47 (9)	C37—C32—C33	115.7 (3)
C31—Sn2—N2	95.38 (11)	C37—C32—C31	123.0 (3)
C24—Sn2—N2	90.89 (12)	C33—C32—C31	121.2 (3)
O2—Sn2—Cl4	85.07 (7)	C34—C33—C32	122.1 (3)
C31—Sn2—Cl4	91.48 (9)	C34—C33—H33	119.0
C24—Sn2—Cl4	95.31 (10)	C32—C33—H33	119.0
N2—Sn2—Cl4	160.53 (7)	C33—C34—C35	119.9 (3)
O3—Sn3—C47	109.91 (11)	C33—C34—H34	120.0
O3—Sn3—C54	112.76 (11)	C35—C34—H34	120.0
C47—Sn3—C54	136.71 (13)	C36—C35—C34	120.1 (4)
O3—Sn3—N3	75.07 (9)	C36—C35—H35	120.0
C47—Sn3—N3	93.83 (11)	C34—C35—H35	120.0
C54—Sn3—N3	90.22 (11)	C35—C36—C37	118.8 (3)
O3—Sn3—Cl7	87.28 (6)	C35—C36—H36	120.6
C47—Sn3—Cl7	92.43 (9)	C37—C36—H36	120.6
C54—Sn3—Cl7	96.48 (10)	C36—C37—C32	123.4 (3)
N3—Sn3—Cl7	162.35 (7)	C36—C37—Cl5	118.1 (3)
C22—O1—Sn1	119.21 (19)	C32—C37—Cl5	118.5 (3)
C45—O2—Sn2	119.16 (19)	N2—C38—C39	122.3 (3)
C68—O3—Sn3	119.50 (19)	N2—C38—H38	118.8
C15—N1—C23	118.8 (3)	C39—C38—H38	118.8
C15—N1—Sn1	131.2 (2)	C40—C39—C38	119.2 (3)
C23—N1—Sn1	110.0 (2)	C40—C39—H39	120.4
C38—N2—C46	119.4 (3)	C38—C39—H39	120.4
C38—N2—Sn2	131.1 (2)	C39—C40—C41	120.4 (3)
C46—N2—Sn2	109.5 (2)	C39—C40—H40	119.8
C61—N3—C69	119.3 (3)	C41—C40—H40	119.8
C61—N3—Sn3	131.3 (2)	C42—C41—C40	125.1 (3)
C69—N3—Sn3	109.4 (2)	C42—C41—C46	118.3 (3)
C2—C1—Sn1	118.5 (2)	C40—C41—C46	116.5 (3)
C2—C1—H1A	107.7	C43—C42—C41	119.8 (3)
Sn1—C1—H1A	107.7	C43—C42—H42	120.1
C2—C1—H1B	107.7	C41—C42—H42	120.1
Sn1—C1—H1B	107.7	C42—C43—C44	121.9 (3)
H1A—C1—H1B	107.1	C42—C43—H43	119.1
C7—C2—C3	116.3 (3)	C44—C43—H43	119.1
C7—C2—C1	123.6 (3)	C45—C44—C43	120.2 (3)
C3—C2—C1	120.0 (3)	C45—C44—H44	119.9
C4—C3—C2	122.3 (4)	C43—C44—H44	119.9
C4—C3—H3	118.8	O2—C45—C44	122.2 (3)
C2—C3—H3	118.8	O2—C45—C46	119.2 (3)
C5—C4—C3	120.2 (4)	C44—C45—C46	118.6 (3)
C5—C4—H4	119.9	N2—C46—C45	116.7 (3)
C3—C4—H4	119.9	N2—C46—C41	122.1 (3)
C4—C5—C6	120.0 (4)	C45—C46—C41	121.2 (3)
C4—C5—H5	120.0	C48—C47—Sn3	116.1 (2)
C6—C5—H5	120.0	C48—C47—H47A	108.3
C7—C6—C5	118.8 (4)	Sn3—C47—H47A	108.3
C7—C6—H6	120.6	C48—C47—H47B	108.3
C5—C6—H6	120.6	Sn3—C47—H47B	108.3
C6—C7—C2	122.3 (3)	H47A—C47—H47B	107.4
C6—C7—Cl2	118.4 (3)	C49—C48—C53	117.4 (3)
C2—C7—Cl2	119.3 (3)	C49—C48—C47	119.6 (3)
C9—C8—Sn1	117.9 (2)	C53—C48—C47	123.0 (3)
C9—C8—H8A	107.8	C50—C49—C48	121.0 (3)
Sn1—C8—H8A	107.8	C50—C49—H49	119.5
C9—C8—H8B	107.8	C48—C49—H49	119.5
Sn1—C8—H8B	107.8	C51—C50—C49	120.2 (4)
H8A—C8—H8B	107.2	C51—C50—H50	119.9
C14—C9—C10	116.2 (3)	C49—C50—H50	119.9
C14—C9—C8	122.4 (3)	C50—C51—C52	120.5 (3)
C10—C9—C8	121.3 (3)	C50—C51—H51	119.7
C11—C10—C9	121.3 (3)	C52—C51—H51	119.7
C11—C10—H10	119.3	C51—C52—C53	118.6 (3)
C9—C10—H10	119.3	C51—C52—H52	120.7
C12—C11—C10	120.4 (3)	C53—C52—H52	120.7
C12—C11—H11	119.8	C52—C53—C48	122.3 (3)
C10—C11—H11	119.8	C52—C53—Cl8	118.5 (3)
C13—C12—C11	119.7 (3)	C48—C53—Cl8	119.2 (3)
C13—C12—H12	120.2	C55—C54—Sn3	113.5 (2)
C11—C12—H12	120.2	C55—C54—H54A	108.9
C12—C13—C14	119.0 (3)	Sn3—C54—H54A	108.9
C12—C13—H13	120.5	C55—C54—H54B	108.9
C14—C13—H13	120.5	Sn3—C54—H54B	108.9
C13—C14—C9	123.3 (3)	H54A—C54—H54B	107.7
C13—C14—Cl3	118.6 (2)	C60—C55—C56	115.9 (3)
C9—C14—Cl3	118.1 (3)	C60—C55—C54	121.9 (3)
N1—C15—C16	122.4 (3)	C56—C55—C54	122.1 (3)
N1—C15—H15	118.8	C57—C56—C55	121.7 (3)
C16—C15—H15	118.8	C57—C56—H56	119.1
C17—C16—C15	119.4 (3)	C55—C56—H56	119.1
C17—C16—H16	120.3	C56—C57—C58	120.5 (3)
C15—C16—H16	120.3	C56—C57—H57	119.8
C16—C17—C18	120.2 (3)	C58—C57—H57	119.8
C16—C17—H17	119.9	C57—C58—C59	120.1 (3)
C18—C17—H17	119.9	C57—C58—H58	119.9
C19—C18—C23	118.6 (3)	C59—C58—H58	119.9
C19—C18—C17	125.1 (3)	C60—C59—C58	118.3 (3)
C23—C18—C17	116.4 (3)	C60—C59—H59	120.8
C20—C19—C18	119.9 (3)	C58—C59—H59	120.8
C20—C19—H19	120.1	C59—C60—C55	123.4 (3)
C18—C19—H19	120.1	C59—C60—Cl9	118.3 (3)
C19—C20—C21	121.8 (3)	C55—C60—Cl9	118.2 (3)
C19—C20—H20	119.1	N3—C61—C62	122.1 (3)
C21—C20—H20	119.1	N3—C61—H61	118.9
C22—C21—C20	120.1 (3)	C62—C61—H61	118.9
C22—C21—H21	120.0	C63—C62—C61	119.2 (3)
C20—C21—H21	120.0	C63—C62—H62	120.4
O1—C22—C21	122.1 (3)	C61—C62—H62	120.4
O1—C22—C23	119.2 (3)	C62—C63—C64	120.4 (3)
C21—C22—C23	118.6 (3)	C62—C63—H63	119.8
N1—C23—C18	122.8 (3)	C64—C63—H63	119.8
N1—C23—C22	116.3 (3)	C65—C64—C69	118.6 (3)
C18—C23—C22	121.0 (3)	C65—C64—C63	125.0 (3)
C25—C24—Sn2	117.0 (2)	C69—C64—C63	116.5 (3)
C25—C24—H24A	108.1	C66—C65—C64	119.9 (3)
Sn2—C24—H24A	108.1	C66—C65—H65	120.0
C25—C24—H24B	108.1	C64—C65—H65	120.0
Sn2—C24—H24B	108.1	C65—C66—C67	121.5 (3)
H24A—C24—H24B	107.3	C65—C66—H66	119.2
C30—C25—C26	116.6 (3)	C67—C66—H66	119.2
C30—C25—C24	122.2 (3)	C68—C67—C66	120.3 (3)
C26—C25—C24	121.1 (3)	C68—C67—H67	119.9
C27—C26—C25	120.9 (3)	C66—C67—H67	119.9
C27—C26—H26	119.5	O3—C68—C67	121.9 (3)
C25—C26—H26	119.5	O3—C68—C69	119.5 (3)
C28—C27—C26	121.0 (3)	C67—C68—C69	118.7 (3)
C28—C27—H27	119.5	N3—C69—C64	122.5 (3)
C26—C27—H27	119.5	N3—C69—C68	116.5 (3)
C27—C28—C29	119.5 (3)	C64—C69—C68	121.0 (3)
C27—C28—H28	120.2		
			
C8—Sn1—O1—C22	89.9 (2)	C28—C29—C30—Cl6	179.1 (3)
C1—Sn1—O1—C22	−88.1 (2)	C26—C25—C30—C29	−1.7 (5)
N1—Sn1—O1—C22	0.3 (2)	C24—C25—C30—C29	178.5 (3)
Cl1—Sn1—O1—C22	−178.8 (2)	C26—C25—C30—Cl6	−179.5 (2)
C31—Sn2—O2—C45	−91.2 (2)	C24—C25—C30—Cl6	0.7 (4)
C24—Sn2—O2—C45	85.1 (2)	O2—Sn2—C31—C32	92.9 (3)
N2—Sn2—O2—C45	−0.7 (2)	C24—Sn2—C31—C32	−81.6 (3)
Cl4—Sn2—O2—C45	179.0 (2)	N2—Sn2—C31—C32	16.4 (3)
C47—Sn3—O3—C68	91.4 (2)	Cl4—Sn2—C31—C32	178.2 (3)
C54—Sn3—O3—C68	−81.2 (2)	Sn2—C31—C32—C37	72.5 (4)
N3—Sn3—O3—C68	2.7 (2)	Sn2—C31—C32—C33	−110.9 (3)
Cl7—Sn3—O3—C68	−177.0 (2)	C37—C32—C33—C34	1.9 (5)
O1—Sn1—N1—C15	−178.2 (3)	C31—C32—C33—C34	−174.9 (3)
C8—Sn1—N1—C15	73.1 (3)	C32—C33—C34—C35	−1.3 (6)
C1—Sn1—N1—C15	−69.0 (3)	C33—C34—C35—C36	−0.4 (6)
Cl1—Sn1—N1—C15	−175.6 (2)	C34—C35—C36—C37	1.3 (6)
O1—Sn1—N1—C23	−0.01 (19)	C35—C36—C37—C32	−0.6 (5)
C8—Sn1—N1—C23	−108.8 (2)	C35—C36—C37—Cl5	178.2 (3)
C1—Sn1—N1—C23	109.1 (2)	C33—C32—C37—C36	−1.0 (5)
Cl1—Sn1—N1—C23	2.5 (4)	C31—C32—C37—C36	175.8 (3)
O2—Sn2—N2—C38	177.9 (3)	C33—C32—C37—Cl5	−179.7 (3)
C31—Sn2—N2—C38	−72.6 (3)	C31—C32—C37—Cl5	−2.9 (4)
C24—Sn2—N2—C38	68.5 (3)	C46—N2—C38—C39	0.1 (5)
Cl4—Sn2—N2—C38	177.2 (2)	Sn2—N2—C38—C39	−177.2 (2)
O2—Sn2—N2—C46	0.46 (19)	N2—C38—C39—C40	−0.6 (5)
C31—Sn2—N2—C46	109.9 (2)	C38—C39—C40—C41	0.1 (5)
C24—Sn2—N2—C46	−109.0 (2)	C39—C40—C41—C42	−179.6 (3)
Cl4—Sn2—N2—C46	−0.2 (4)	C39—C40—C41—C46	0.6 (5)
O3—Sn3—N3—C61	179.1 (3)	C40—C41—C42—C43	−177.3 (3)
C47—Sn3—N3—C61	69.5 (3)	C46—C41—C42—C43	2.4 (5)
C54—Sn3—N3—C61	−67.4 (3)	C41—C42—C43—C44	−1.2 (5)
Cl7—Sn3—N3—C61	−180.0 (2)	C42—C43—C44—C45	−0.3 (5)
O3—Sn3—N3—C69	−1.12 (19)	Sn2—O2—C45—C44	−179.0 (2)
C47—Sn3—N3—C69	−110.7 (2)	Sn2—O2—C45—C46	0.9 (4)
C54—Sn3—N3—C69	112.4 (2)	C43—C44—C45—O2	−179.5 (3)
Cl7—Sn3—N3—C69	−0.2 (4)	C43—C44—C45—C46	0.5 (5)
O1—Sn1—C1—C2	121.1 (3)	C38—N2—C46—C45	−178.0 (3)
C8—Sn1—C1—C2	−56.0 (4)	Sn2—N2—C46—C45	−0.2 (3)
N1—Sn1—C1—C2	45.5 (3)	C38—N2—C46—C41	0.8 (5)
Cl1—Sn1—C1—C2	−152.5 (3)	Sn2—N2—C46—C41	178.6 (2)
Sn1—C1—C2—C7	92.8 (4)	O2—C45—C46—N2	−0.5 (4)
Sn1—C1—C2—C3	−86.5 (4)	C44—C45—C46—N2	179.5 (3)
C7—C2—C3—C4	−1.5 (5)	O2—C45—C46—C41	−179.2 (3)
C1—C2—C3—C4	177.9 (3)	C44—C45—C46—C41	0.7 (5)
C2—C3—C4—C5	0.6 (6)	C42—C41—C46—N2	179.2 (3)
C3—C4—C5—C6	0.6 (6)	C40—C41—C46—N2	−1.1 (5)
C4—C5—C6—C7	−0.8 (6)	C42—C41—C46—C45	−2.2 (5)
C5—C6—C7—C2	−0.2 (5)	C40—C41—C46—C45	177.6 (3)
C5—C6—C7—Cl2	178.2 (3)	O3—Sn3—C47—C48	−74.1 (2)
C3—C2—C7—C6	1.3 (5)	C54—Sn3—C47—C48	95.8 (3)
C1—C2—C7—C6	−178.1 (3)	N3—Sn3—C47—C48	1.4 (2)
C3—C2—C7—Cl2	−177.1 (3)	Cl7—Sn3—C47—C48	−162.1 (2)
C1—C2—C7—Cl2	3.5 (5)	Sn3—C47—C48—C49	87.5 (3)
O1—Sn1—C8—C9	−98.9 (2)	Sn3—C47—C48—C53	−91.6 (3)
C1—Sn1—C8—C9	78.1 (3)	C53—C48—C49—C50	−0.2 (5)
N1—Sn1—C8—C9	−23.0 (3)	C47—C48—C49—C50	−179.3 (3)
Cl1—Sn1—C8—C9	174.5 (2)	C48—C49—C50—C51	0.9 (6)
Sn1—C8—C9—C14	−69.7 (4)	C49—C50—C51—C52	−1.2 (6)
Sn1—C8—C9—C10	111.7 (3)	C50—C51—C52—C53	0.6 (6)
C14—C9—C10—C11	−1.1 (5)	C51—C52—C53—C48	0.2 (5)
C8—C9—C10—C11	177.6 (3)	C51—C52—C53—Cl8	−179.7 (3)
C9—C10—C11—C12	0.7 (6)	C49—C48—C53—C52	−0.4 (5)
C10—C11—C12—C13	−0.4 (6)	C47—C48—C53—C52	178.7 (3)
C11—C12—C13—C14	0.5 (5)	C49—C48—C53—Cl8	179.5 (2)
C12—C13—C14—C9	−0.9 (5)	C47—C48—C53—Cl8	−1.4 (4)
C12—C13—C14—Cl3	178.5 (3)	O3—Sn3—C54—C55	138.4 (2)
C10—C9—C14—C13	1.2 (5)	C47—Sn3—C54—C55	−31.3 (3)
C8—C9—C14—C13	−177.4 (3)	N3—Sn3—C54—C55	64.5 (2)
C10—C9—C14—Cl3	−178.2 (2)	Cl7—Sn3—C54—C55	−131.8 (2)
C8—C9—C14—Cl3	3.2 (4)	Sn3—C54—C55—C60	68.4 (4)
C23—N1—C15—C16	−0.2 (5)	Sn3—C54—C55—C56	−110.4 (3)
Sn1—N1—C15—C16	177.8 (2)	C60—C55—C56—C57	−0.8 (5)
N1—C15—C16—C17	0.9 (5)	C54—C55—C56—C57	178.0 (3)
C15—C16—C17—C18	−0.7 (5)	C55—C56—C57—C58	−0.5 (5)
C16—C17—C18—C19	−179.1 (3)	C56—C57—C58—C59	1.2 (5)
C16—C17—C18—C23	−0.3 (5)	C57—C58—C59—C60	−0.5 (5)
C23—C18—C19—C20	−1.4 (5)	C58—C59—C60—C55	−0.9 (5)
C17—C18—C19—C20	177.5 (3)	C58—C59—C60—Cl9	180.0 (3)
C18—C19—C20—C21	0.4 (5)	C56—C55—C60—C59	1.5 (5)
C19—C20—C21—C22	0.9 (5)	C54—C55—C60—C59	−177.3 (3)
Sn1—O1—C22—C21	178.7 (2)	C56—C55—C60—Cl9	−179.3 (2)
Sn1—O1—C22—C23	−0.6 (4)	C54—C55—C60—Cl9	1.8 (4)
C20—C21—C22—O1	179.5 (3)	C69—N3—C61—C62	−0.4 (5)
C20—C21—C22—C23	−1.1 (5)	Sn3—N3—C61—C62	179.3 (2)
C15—N1—C23—C18	−0.8 (5)	N3—C61—C62—C63	0.5 (5)
Sn1—N1—C23—C18	−179.2 (2)	C61—C62—C63—C64	−0.4 (5)
C15—N1—C23—C22	178.1 (3)	C62—C63—C64—C65	−178.2 (3)
Sn1—N1—C23—C22	−0.3 (3)	C62—C63—C64—C69	0.3 (5)
C19—C18—C23—N1	179.9 (3)	C69—C64—C65—C66	−1.2 (5)
C17—C18—C23—N1	1.0 (5)	C63—C64—C65—C66	177.2 (3)
C19—C18—C23—C22	1.1 (5)	C64—C65—C66—C67	1.6 (5)
C17—C18—C23—C22	−177.8 (3)	C65—C66—C67—C68	0.1 (5)
O1—C22—C23—N1	0.6 (4)	Sn3—O3—C68—C67	175.5 (2)
C21—C22—C23—N1	−178.8 (3)	Sn3—O3—C68—C69	−4.0 (4)
O1—C22—C23—C18	179.5 (3)	C66—C67—C68—O3	178.4 (3)
C21—C22—C23—C18	0.2 (4)	C66—C67—C68—C69	−2.0 (5)
O2—Sn2—C24—C25	−139.5 (2)	C61—N3—C69—C64	0.3 (5)
C31—Sn2—C24—C25	35.0 (4)	Sn3—N3—C69—C64	−179.5 (2)
N2—Sn2—C24—C25	−64.6 (3)	C61—N3—C69—C68	179.4 (3)
Cl4—Sn2—C24—C25	133.9 (3)	Sn3—N3—C69—C68	−0.4 (3)
Sn2—C24—C25—C30	−80.0 (4)	C65—C64—C69—N3	178.4 (3)
Sn2—C24—C25—C26	100.2 (3)	C63—C64—C69—N3	−0.2 (5)
C30—C25—C26—C27	0.8 (5)	C65—C64—C69—C68	−0.7 (5)
C24—C25—C26—C27	−179.4 (3)	C63—C64—C69—C68	−179.3 (3)
C25—C26—C27—C28	0.4 (5)	O3—C68—C69—N3	2.8 (4)
C26—C27—C28—C29	−0.9 (6)	C67—C68—C69—N3	−176.8 (3)
C27—C28—C29—C30	0.1 (5)	O3—C68—C69—C64	−178.1 (3)
C28—C29—C30—C25	1.2 (5)	C67—C68—C69—C64	2.3 (5)
